# Subcellular Detection of SARS-CoV-2 RNA in Human Tissue Reveals Distinct Localization in Alveolar Type 2 Pneumocytes and Alveolar Macrophages

**DOI:** 10.1128/mbio.03751-21

**Published:** 2022-02-08

**Authors:** Kofi K. Acheampong, Dylan L. Schaff, Benjamin L. Emert, Jonathan Lake, Sam Reffsin, Emily K. Shea, Courtney E. Comar, Leslie A. Litzky, Nigar A. Khurram, Rebecca L. Linn, Michael Feldman, Susan R. Weiss, Kathleen T. Montone, Sara Cherry, Sydney M. Shaffer

**Affiliations:** a Department of Pathology and Laboratory Medicine, Perelman School of Medicine, University of Pennsylvania, Philadelphia, Pennsylvania, USA; b Department of Bioengineering, School of Engineering and Applied Science, University of Pennsylvania, Philadelphia, Pennsylvania, USA; c Genomics and Computational Biology Graduate Group, Perelman School of Medicine, University of Pennsylvania, Philadelphia, Pennsylvania, USA; d Department of Cancer Biology, Perelman School of Medicine, University of Pennsylvania, Philadelphia, Pennsylvania, USA; e Department of Microbiology, University of Pennsylvania, Philadelphia, Pennsylvania, USA; f Penn Center for Research on Coronaviruses and Other Emerging Pathogens, Philadelphia, Pennsylvania, USA; g Department of Pathology, Northwestern University, Feinberg School of Medicine, Chicago, Illinois, USA; h Division of Anatomic Pathology, The Children’s Hospital of Philadelphia, Philadelphia, Pennsylvania, USA; i Department of Biochemistry and Biophysics, University of Pennsylvania, Philadelphia, Pennsylvania, USA; j Clinical Microbiology Laboratory, Hospital of the University of Pennsylvania, Philadelphia, Pennsylvania, USA; k Infectious Disease Diagnostics Laboratory, The Children’s Hospital of Philadelphia, Philadelphia, Pennsylvania, USA; Rutgers-Robert Wood Johnson Medical School

**Keywords:** RNA FISH, cellular imaging, fluorescent image analysis, single cell

## Abstract

The widespread coronavirus disease 2019 (COVID-19) is caused by infection with the novel coronavirus SARS-CoV-2. Currently, we have limited understanding of which cells become infected with SARS-CoV-2 in human tissues and where viral RNA localizes on the subcellular level. Here, we present a platform for preparing autopsy tissue for visualizing SARS-CoV-2 RNA using RNA fluorescence in situ hybridization (FISH) with amplification by hybridization chain reaction. We developed probe sets that target different regions of SARS-CoV-2 (including ORF1a and N), as well as probe sets that specifically target SARS-CoV-2 subgenomic mRNAs. We validated these probe sets in cell culture and tissues (lung, lymph node, and placenta) from infected patients. Using this technology, we observe distinct subcellular localization patterns of the ORF1a and N regions. In human lung tissue, we performed multiplexed RNA FISH HCR for SARS-CoV-2 and cell-type-specific marker genes. We found viral RNA in cells containing the alveolar type 2 (AT2) cell marker gene (*SFTPC*) and the alveolar macrophage marker gene (*MARCO*) but did not identify viral RNA in cells containing the alveolar type 1 (AT1) cell marker gene (*AGER*). Moreover, we observed distinct subcellular localization patterns of viral RNA in AT2 cells and alveolar macrophages. In sum, we demonstrate the use of RNA FISH HCR for visualizing different RNA species from SARS-CoV-2 in cell lines and FFPE (formalin fixation and paraffin embedding) autopsy specimens. We anticipate that this platform could be broadly useful for studying SARS-CoV-2 pathology in tissues, as well as extended for other applications, including investigating the viral life cycle, viral diagnostics, and drug screening.

## INTRODUCTION

The ongoing coronavirus disease 2019 (COVID-19) pandemic is caused by the betacoronavirus, severe acute respiratory syndrome coronavirus 2 (SARS-CoV-2) (1). COVID-19 manifests in a highly variable manner from person to person, with some infected individuals being completely asymptomatic, while others experience symptoms ranging from mild upper respiratory disease to severe pneumonia to multiorgan failure (1–3). With such a diverse array of disease symptoms, characterizing the distribution of the SARS-CoV-2 virus across various human tissues is crucial to improving our understanding of COVID-19 pathogenesis, pathophysiology, and identifying and rationally designing effective therapies.

Critical to defining the distribution of the SARS-CoV-2 virus in humans is determining which organs and cell types become infected with SARS-CoV-2. Several studies (4–9) have made predictions on the tissues and cell types infected by SARS-CoV-2 based on host expression of factors known to facilitate viral entry into the host cell for closely related betacoronaviruses such as SARS-CoV-1 and MERS-CoV. While this approach has helped to narrow down targets in humans, the confirmation of these predicted organs and cell types as true targets of SARS-CoV-2 remains an ongoing process. Accordingly, in a limited set of human autopsy studies, SARS-CoV-2 components (RNA and proteins) have been detected in multiple organs and organ systems, including the upper airway (10, 11), lung (11, 12), gastrointestinal tract (13), placenta (14, 15), spleen (16), myocardium (17), and lymph node (16), using different combinations of RT-PCR, immunostaining, electron microscopy, and *in situ* hybridization techniques. A number of these studies that use techniques with single-cell resolution (such as immunostaining and *in situ* hybridization) have identified alveolar type 2 (AT2) cells and alveolar macrophages in the lung (11), glandular epithelial cells in the gastrointestinal tract (13), cytotrophoblasts and syncytiotrophoblasts in the placenta (18), and macrophages in the spleen and lymph nodes (16) as specific cell types containing SARS-CoV-2 viral components. Ultimately, defining the full range of SARS CoV-2 tropism requires direct detection approaches to validate the predictions from bioinformatic analyses in large sets of human tissue samples from COVID-19 patients.

Similarly, our current knowledge about viral life cycle and sites of SARS CoV-2 RNA synthesis is incomplete and largely based upon studies from SARS CoV-1 and MERS CoV. Upon cellular infection with these viruses, one of the early stages of the viral life cycle is assembly of the replication/transcription complex (RTC), which is the site of both viral replication and transcription of subgenomic mRNAs. Furthermore, coronaviruses both replicate their genomic RNA and transcribe subgenomic mRNAs. The subgenomic mRNAs are generated by discontinuous transcription that generates transcripts containing a conserved upstream leader sequence and a downstream body encoding viral proteins (19). While these transcripts have been documented by Northern blotting (for 40 years) and captured with sequencing techniques, they are yet to be directly visualized *in situ* in single cells.

RNA fluorescence *in situ* hybridization (FISH) techniques are ideal to address questions about both the cell types infected by SARS-CoV-2 and the subcellular localization of viral transcripts. Previously, robust RNA *in situ* assays have been developed for a number of different viral targets (20–22). However, most human specimens from COVID patients are preserved with formalin fixation and paraffin embedding (FFPE) for long-term storage and biosafety. FFPE preserved tissues are not well suited for single-molecule RNA FISH since the probes generate relatively low signal and the tissues have substantial autofluorescence. Furthermore, single-molecule RNA FISH probes require large regions of unique target sequence and thus are not amenable to specifically targeting subgenomic mRNAs, which have largely the same sequence as the genomic transcripts.

Here, we present a platform and methodology for addressing these emerging questions about SARS-CoV-2 subcellular localization and cellular tropism using RNA FISH. We leverage single-molecule RNA FISH (23) and the signal amplification capabilities of hybridization chain reaction v3.0 (HCR) (24) to image different SARS-CoV-2 viral RNA species in cell culture infection models and FFPE human autopsy specimens. We extend the assay to multiplex probe sets for viral and host RNAs to simultaneously detect cells with viral RNA and determine their cell type. This platform allows us to observe differences in RNA staining patterns of SARS-CoV-2 infection between AT2 cells and alveolar macrophages in human lung autopsy tissue.

## RESULTS

**FIG 1:**
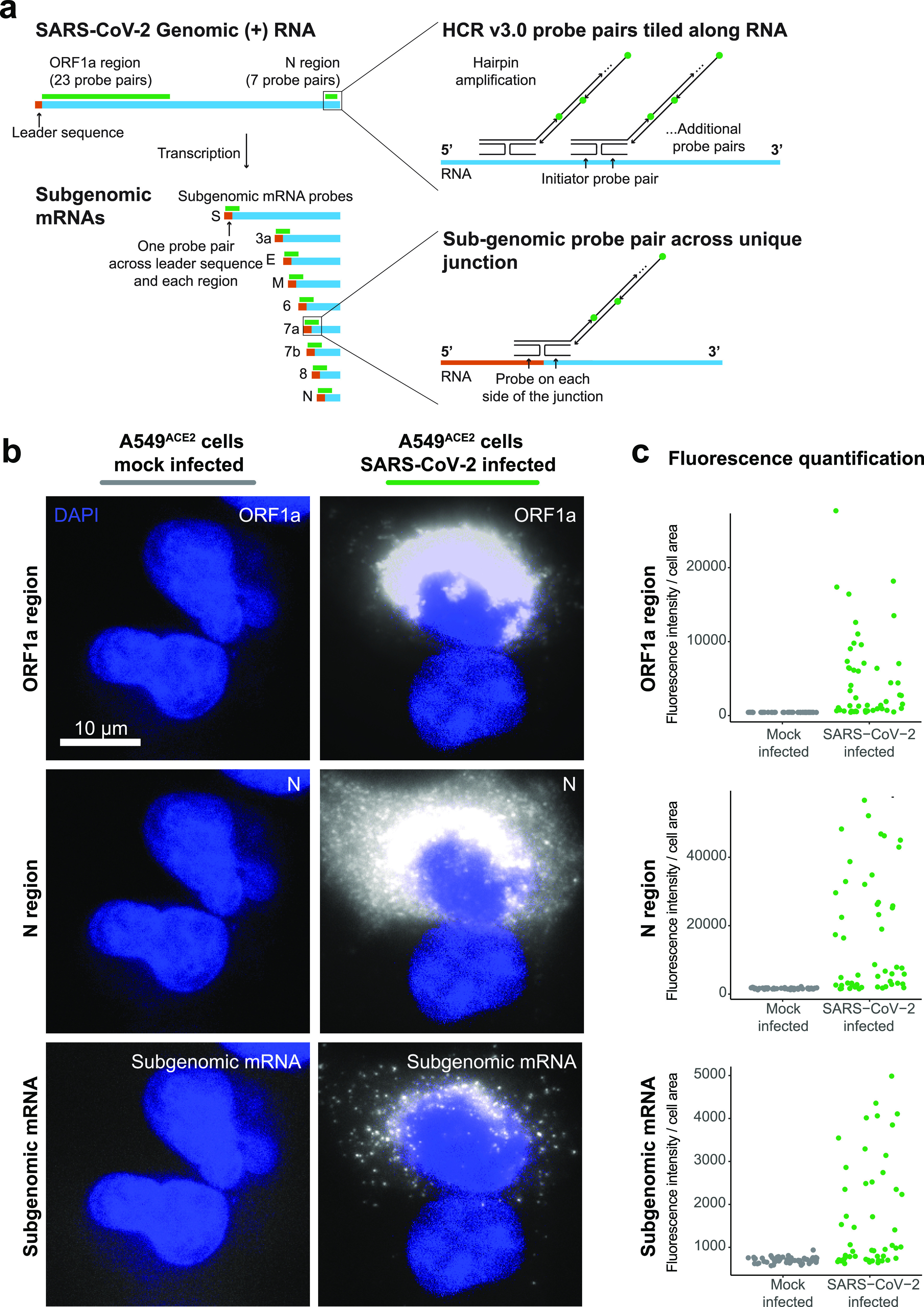
RNA FISH HCR v3.0 probe sets enable direct visualization of the SARS-CoV-2 virus. (a) Schematic of the SARS-CoV-2 genomic RNA and subgenomic RNA species with HCR v3.0 probe designs highlighted. We developed probes tiled along the ORF1a and N regions of the SARS-CoV-2 (+) RNA strand. These probe sets consisted of 23 probe pairs for ORF1a and 7 probe pairs for N. To detect all of the subgenomic RNAs, we positioned the HCR probes across the junction of the leader sequence and each unique subgenomic transcript. In the schematic, the leader sequence is shown in orange, the transcript is shown in blue, and the probe design is shown in green. (b) Representative images of the A549^ACE2^ cells mock infected or infected with SARS-CoV-2 at an MOI = 1, fixed 24 h postinfection, and then hybridized with probes for ORF1a, N, and subgenome. DAPI labels cell nuclei. The scale bar applies to all images and shows 10 μm. The images are z-projections from image stacks acquired at ×60 magnification. (c) Quantification of the fluorescence signal from the experiment in panel b. For each the mock-infected data set (shown in gray) and SARS-CoV-2-infected data set (shown in green), we quantified fluorescence signal intensity from 50 cells per condition. We found that for each probe set the SARS-CoV-2-infected sample had statistically significant differences in the distribution of fluorescence intensities compared to the mock-infected sample (ORF1a region, N region, and subgenomic RNAs, single-tailed KS test *P* values = 4.765e–16, <2.2e–16, and 4.496e–09, respectively). Note that the SARS-CoV-2-infected sample contained both cells that were infected and cells that remained uninfected.

RNA *in situ* hybridization technologies offer the ability to visualize RNA within fixed cells and tissues. Such technologies have been used for both cellular RNAs and viral RNAs in infected cells (21–23, 25, 26). With the emergence of the SARS-CoV-2 virus, we sought to use RNA *in situ* hybridization techniques to visualize the viral RNA transcripts in both cell lines and tissues. To overcome limitations from background autofluorescence and for robust RNA detection, we used hybridization chain reaction v3.0 (HCR) to achieve amplification of the RNA FISH signal (24). We developed probe sets consisting of multiple probe pairs that are tiled along the RNA sequence of interest. Each probe pair, termed “split-initiator” probes, contains a region of complementarity to the viral RNA and half of the initiator sequence for signal amplification via polymerization of dye-conjugated DNA hairpins. Because the initiator sequence is divided between the two split-initiator probes, amplification only occurs if both of the probes bind adjacently, providing additional specificity for the target of interest (Fig. 1a). As previously described (24), we used a two-stage protocol in which we first hybridize the split-initiator probes and then amplify the signal using fluorescently labeled DNA hairpins. We can multiplex the assay for multiple targets by using distinct hairpin sequences labeled with different fluorophores for each RNA target.

We first designed probe sets targeting the positive stranded SARS-CoV-2 RNA sequences of the ORF1a and N regions (Fig. 1a). We tested these probe sets in A549^ACE2^ human lung cancer cells infected with SARS-CoV-2 at a multiplicity of infection (MOI) of 1, as well as mock-infected A549^ACE2^ cells. We found high-intensity fluorescent labeling with both the ORF1a and N probe sets in the infected but not in mock-infected samples (Fig. 1b). Staining from both probe sets was confined to the cytoplasm of cells and did not stain inside the cell nucleus. Interestingly, we observed that the ORF1a probe set (which labels only genomic RNA) showed the highest intensity of staining in a region around the periphery of the nucleus of each cell. This staining pattern is consistent with reported coronavirus RNA replication at replication/transcription complexes (RTCs), which are networks consisting of host endoplasmic reticulum (ER)-derived, perinuclear, double-membrane structures (27–29). Meanwhile, the N region probe set showed more diffuse staining throughout the cytoplasm but higher intensity in the perinuclear region. Such a pattern could be expected for the N region probes since they are likely binding both genomic RNA species in the RTCs and all the subgenomic mRNA species (Fig. 1a). These subgenomic mRNAs are translated by the host ribosome and thus are more diffuse through the cytoplasm rather than largely confined to viral replication centers.

To further resolve the localization of genomic and subgenomic mRNA, we designed probes to uniquely label the subgenomic mRNA species without simultaneously targeting SARS-CoV-2 genomic RNA. Such a probe design is difficult because the subgenomic mRNA sequence is also contained within the genomic RNA, and thus subgenomic and genomic transcripts would be simultaneously targeted with conventional probe designs. Thus, to develop subgenome-specific probes, we leveraged a feature of coronavirus transcription biology. To generate subgenomic mRNAs, the viral polymerase first transcribes negative-strand intermediates from which it then transcribes the subgenomic mRNAs. During this synthesis of negative-strand intermediates, the polymerase terminates transcription when it encounters transcription regulatory sequences (TRSs) upstream of each subgenomic mRNA open reading frame and resumes at a TRS located further toward the 5′ end of the genomic template. This interrupted form of transcription, known as discontinuous transcription (19, 30), adds an antisense copy of the genomic leader sequence to each subgenomic mRNA intermediate. Therefore, in the subgenomic mRNAs only, there is a unique junction formed between the 3′ end of the leader sequence and the 5′ end of their gene sequence. To target each individual subgenomic mRNA, we designed HCR probe pairs that span the unique junction sites, with one of the split-initiator probes positioned on the leader sequence and the other split-initiator probe on the gene sequence (Fig. 1a). Because each split-initiator probe contains half the initiator sequence, amplification should only occur if the two probes bind adjacent to each other, which would be the case for each target subgenomic mRNA but not genomic RNA. With this strategy, we achieve highly specific detection of the fusion transcripts containing the leader and each subgenomic sequence. We designed these subgenomic probes for each of the eight different canonical subgenomic mRNA species and used them together on A549^ACE2^ cells infected with SARS-CoV-2 (Fig. 1b). We found that the subgenomic mRNA probe sets showed a diffuse staining pattern throughout the cytoplasm that is distinct from both the ORF1a and N probe sets. This diffuse staining pattern is consistent with the subgenomic mRNAs being distributed throughout the cytoplasm for translation by free ribosomes or ER-associated ribosomes (rather than concentrated in the replication/transcription complex as seen for the ORF1a probe set).

Next, we quantified the fluorescence intensity from each of these probe sets in both the infected and mock-infected cells. We found that the mean fluorescence intensity of infected cells was significantly higher than the fluorescence intensity of uninfected cells in the mock-infected sample (Fig. 1c). Of note, the infected samples contained a mixture of infected and noninfected cells, which are clearly distinguishable *in situ*. We also designed conventional (nonamplified) single-molecule RNA FISH probes to the same regions of SARS-CoV-2 genomic RNA and compared the signal from the amplified RNA FISH HCR to the nonamplified single-molecule RNA FISH (see Fig. S1). We again found with single-molecule RNA FISH that the ORF1a probe set had a perinuclear staining pattern, while the N probe had a more diffuse cytoplasmic distribution. Compared to nonamplified single-molecule RNA FISH, the RNA FISH HCR signal was significantly brighter, requiring much shorter exposure times for imaging (50- to 100-ms exposure times for RNA FISH HCR compared to 300- to 500-ms exposure times for single-molecule RNA FISH). Thus, we selected RNA FISH HCR for subsequent experiments in tissues in which we expected higher background compared to cell culture conditions.

10.1128/mbio.03751-21.1 FIG S1 Single-molecule RNA FISH with probes targeting ORF1a and N regions in Huh7.5 cells infected with SARS-CoV-2. Representative images of Huh7.5 cells infected with SARS-CoV-2 and hybridized with RNA FISH probes targeting ORF1a and N are shown. Similar to the RNA FISH HCR, we observed higher fluorescence signal intensity in the perinuclear region of the ORF1a probe compared to the N probe. The bottom row of images is a composite of the ORF1a probe (green), N probe (red), and DAPI signal. In all images, the DAPI stain for cell nuclei is shown in blue. Scale bars are 10 μm. The images are z-projections from image stacks acquired at 60×. Download FIG S1, PDF file, 2.7 MB. Copyright © 2022 Acheampong et al. 2022 Acheampong et al. https://creativecommons.org/licenses/by/4.0/ This content is distributed under the terms of the Creative Commons Attribution 4.0 International license.

**FIG 2:**
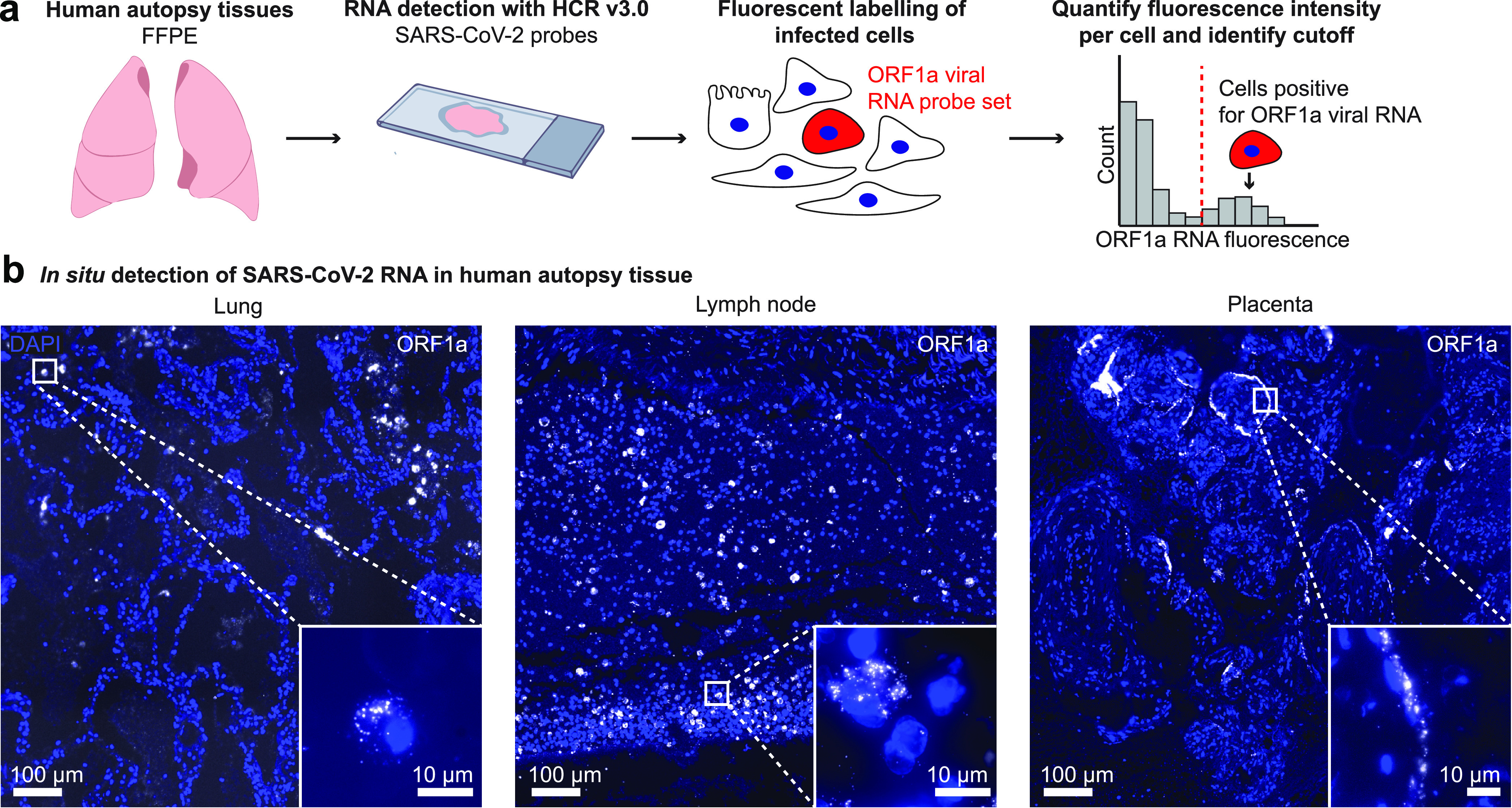
RNA FISH HCR in FFPE human autopsy tissues. (a) Experiment design in which we performed RNA FISH HCR with ORF1a probe sets on FFPE tissues including lung, hilar lymph node, and placenta. (b) Example images of each tissue with ORF1a RNA staining. Images are large area scans of image tiles acquired at 20×. The scale bar on the large images shows 100 μm. Inset images show a zoomed in example of ORF1a RNA staining in that tissue. Scale bars on these inset images are 10 μm. DAPI stain (blue) labels the cell nuclei in all images.

We next sought to use this assay in human tissues to localize sites of SARS-CoV-2 viral RNA. We analyzed human autopsy specimens from research autopsies of COVID-19 patients performed at the University of Pennsylvania and Children’s Hospital of Philadelphia in 2020. The tissues were fixed in neutral buffered formalin, embedded in paraffin, sectioned, and then probed for SARS-CoV-2 RNA (Fig. 2a). We analyzed a total of 14 different lung specimens from eight patients and found one case that showed extensive staining of SARS-CoV-2 RNA in lung tissue (see Table S1). Of note, the patient with extensive virus staining in the lung was immunosuppressed and decompensated within 2 days of arriving at the hospital. In this specimen, we observed discrete regions of the lung containing infected cells (Fig. 2b, lung), as well as regions with bright fluorescence signal, but no nuclei present (see Fig. S2a). To find areas with SARS-CoV-2 staining, we developed a computational pipeline (described in Materials and Methods) to segment cells and then quantify the fluorescence staining from the ORF1a viral probe set (output of the analysis is shown in Fig. S3). As a control, we also examined lung tissue from patients who were not infected with SARS-CoV-2. In these controls, our computational analysis did not identify any cells passing the threshold of significant ORF1a viral RNA staining (see Fig. S4).

10.1128/mbio.03751-21.2 FIG S2 Examples of regions in the lung tissue that show viral staining with ORF1a probes, but do not have nuclei. (a) Two example regions in which we observed extensive viral staining with the ORF1a probe set but did not observe DAPI signal. The right image is RNA FISH HCR for ORF1a, and the left image is a bright-field image. Red dotted lines show areas of interest with ORF1a staining. (b) Examples of small discrete regions of ORF1a staining without DAPI staining. In all images, the DAPI stain for cell nuclei is shown in blue. Scale bars indicate 10 μm. The images are z-projections of image stacks acquired at 100× magnification. Download FIG S2, PDF file, 0.2 MB. Copyright © 2022 Acheampong et al. 2022 Acheampong et al. https://creativecommons.org/licenses/by/4.0/ This content is distributed under the terms of the Creative Commons Attribution 4.0 International license.

10.1128/mbio.03751-21.3 FIG S3 Computational analysis identifying suspected infected cells in each tissue. Images overlayed with the results from our image processing pipeline. Blue dots label cells with fluorescence signal below the cutoff for infected, and red dots label cells above the cutoff for infection. Images are from a subset of the data. The total number of cells are displayed in each region, and the total number of cells in each data set is below each image. Images are large area scans of individual image tiles acquired at 20× magnification. Download FIG S3, PDF file, 0.3 MB. Copyright © 2022 Acheampong et al. 2022 Acheampong et al. https://creativecommons.org/licenses/by/4.0/ This content is distributed under the terms of the Creative Commons Attribution 4.0 International license.

10.1128/mbio.03751-21.4 FIG S4 Comparison of infected tissue to control tissues samples in which the patients did not have SARS-CoV-2 infection. We performed RNA FISH HCR in lung (a) and placenta (b) samples from patients with SARS-CoV-2 infection (yellow) and control samples from patients that did not have SARS-CoV-2 infection (purple and teal). Histograms display the log_2_ transformation of median normalized ORF1a fluorescence signal in each cell. The distribution of median-normalized ORF1a fluorescence signal is significantly different in infected samples compared to control samples where the infected samples have a tail made up of infected cells with much higher signal than control tissue in both the lung (single-tailed KS test, *P* < 2.2e–16 [compared to control sample 1] and *P* < 2.2e–16 [compared to control sample 2]) and the placenta (single-tailed KS test, *P* < 2.2e–16). Download FIG S4, PDF file, 0.5 MB. Copyright © 2022 Acheampong et al. 2022 Acheampong et al. https://creativecommons.org/licenses/by/4.0/ This content is distributed under the terms of the Creative Commons Attribution 4.0 International license.

10.1128/mbio.03751-21.8 TABLE S1 COVID tissues evaluated using RNA FISH HCR. The table summarizes the different tissues and patient numbers from the COVID autopsy series. Download Table S1, XLSX file, 0.01 MB. Copyright © 2022 Acheampong et al. 2022 Acheampong et al. https://creativecommons.org/licenses/by/4.0/ This content is distributed under the terms of the Creative Commons Attribution 4.0 International license.

From the same patient with extensive lung infection, we surveyed other tissues for SARS-CoV-2 RNA. We performed RNA FISH HCR with the ORF1a virus probes on a total of 11 different tissues including esophagus, kidney, liver, hilar lymph node, spleen, heart, stomach, ileum, duodenum, jejunum, and trachea. Of all of these tissues, we only detected viral RNA with our probe sets in the hilar lymph node. Of note, it is possible that some of the other tissues from this patient also contained virus but underwent more degradation prior to RNA FISH HCR. In the lymph node specimen, we found ORF1a RNA FISH HCR signal localized to cells scattered throughout the tissue (Fig. 2b). These results are consistent with other studies reporting the detection of SARS-CoV-2 nucleocapsid-positive cells in hilar lymph nodes (31, 32).

We also analyzed two human placenta samples from cases in which the mother tested positive for SARS-CoV-2. Both samples showed cells with ORF1a probe set staining localized predominantly along the periphery of villi structures (Fig. 2b, placenta). This pattern is consistent with other reports using immunohistochemical assays, electron microscopy, and RNAscope *in situ* hybridization (18, 33–35) that show viral localization to syncytiotrophoblasts, which are located along the villous periphery and interface with maternal blood. We further confirmed this observation through comparison with an adjacent hematoxylin and eosin (H&E)-stained slide (see Fig. S5).

10.1128/mbio.03751-21.5 FIG S5 Example region of placenta with RNA FISH HCR for ORF1a with an adjacent tissue section stained with H&E. We performed RNA FISH HCR with probe sets for ORF1a and *EGFR*. On the adjacent section, we stained the tissue with hematoxylin and eosin. We took tiled image scans of the fluorescence slide and used a slide scanner for the H&E. We aligned the two images to identify the corresponding H&E region for which we found cells staining with the ORF1a probe set. ORF1a fluorescence signal is in pink, *EGFR* is in yellow, and DAPI is in blue. Images are large area scans of image tiles acquired at 20× magnification. Download FIG S5, PDF file, 0.2 MB. Copyright © 2022 Acheampong et al. 2022 Acheampong et al. https://creativecommons.org/licenses/by/4.0/ This content is distributed under the terms of the Creative Commons Attribution 4.0 International license.

After using these probe sets to localize the virus across tissues, we next wanted to know whether multiplexed RNA FISH HCR could be used to determine what cell types become infected with the virus in the lung. We developed a robust strategy for selecting cell-type-specific marker genes for multiplexed *in situ* analysis, along with SARS-CoV-2 RNA. We used single-cell RNA-sequencing data from the human lung atlas (36) to identify genes that would uniquely label alveolar type 1 (AT1) cells, alveolar type 2 (AT2) cells, and alveolar macrophages within the lung. For our analysis, we considered the specificity of the marker gene, the fraction of cells that expressed the marker, and the expression level (as we needed genes with high enough expression for accurate detection *in situ*). We only considered markers that were present in all of the human lung cell atlas subjects to avoid genes with heterogeneous expression between individuals. We developed HCR probes for 1 marker of each cell type (*AGER* for AT1 cells, *SFTPC* for AT2 cells, and *MARCO* for alveolar macrophages; see Fig. S6).

10.1128/mbio.03751-21.6 FIG S6 tSNE plots of the human lung cell atlas single-cell RNA-sequencing data across three subjects. Each plot is a tSNE projection of all cells in the data set, with the color of the points depicting the expression of the gene. Each row of plots is from a different subject. The target cell type with each marker is labeled on the plots with a circle around the cluster (monocytes/macrophages, AT1 cells, and AT2 cells). The three genes identified as cell-type-specific markers are *MARCO*, *AGER*, and *SFTPC*. Download FIG S6, PDF file, 0.7 MB. Copyright © 2022 Acheampong et al. 2022 Acheampong et al. https://creativecommons.org/licenses/by/4.0/ This content is distributed under the terms of the Creative Commons Attribution 4.0 International license.

**FIG 3:**
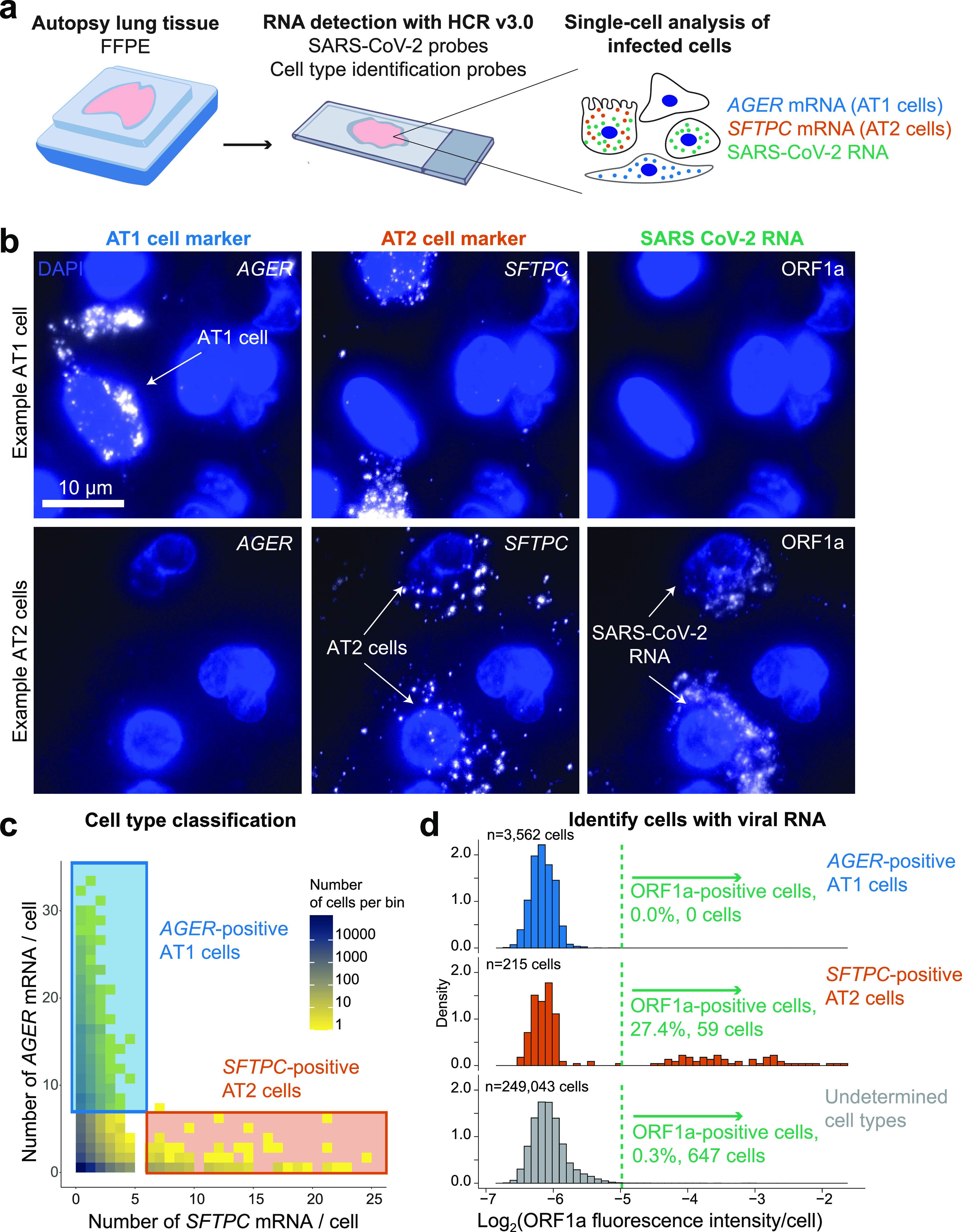
Multiplexed RNA FISH HCR identifies AT2 cells containing viral RNA in lung autopsy samples. (a) We probed FFPE human lung tissue with SARS-CoV-2 probe sets, as well as probe sets for cell-type-specific marker genes, *AGER* for AT1 cells, and *SFTPC* for AT2 cells. (b) Representative images of cells classified as AT1 cells or AT2 cells. The top row depicts an AT1 cell staining positive for *AGER*. The second row shows two *SFTPC*-positive AT2 cells staining with the ORF1a viral RNA probe set. DAPI stain (blue) labels the cell nuclei in all images. Scale bars show 10 μm. The images are z-projections of image stacks acquired at ×100 magnification. (c) Here, we acquired large tiled image scans consisting of 252,820 cells total. We quantified the *AGER* and *SFTPC* mRNA in each cell and set a cutoff (see Materials and Methods) for determining which cells are positive for each gene, indicating that they are either AT1 or AT2 cells, respectively. The plot shows a scatterplot of mRNA levels with cutoffs for AT1 and AT2 cells. The color on the scatterplot indicates the number of cells at each point on the plot, and the scale is shown by the legend with yellow indicating low cell numbers and blue indicating high cell numbers. The blue rectangle shows the region on the plot for *AGER*-positive AT1 cells, and the red rectangle shows the region on the plot for *SFTPC*-positive AT2 cells. (d) Histograms of the log_2_ of the fluorescence intensity for the SARS-CoV-2 ORF1a probe set in each cell. The data are split into three histograms for each cell identified (AT1 cells, AT2 cells, and undetermined cells). These histograms are normalized to the number of cells in each category. The *y* axis labels the density of these distributions (which is the normalized number of cells in each bin). The total number of cells in each category is labeled on the plot. The green dotted line shows the cutoff for calling a cell positive for viral RNA. AT2 cells had a statistically significant different distribution of ORF1a signal compared to AT1 cells (single-tailed KS test, *P* = 2.653e–15).

We first performed multiplexed RNA FISH HCR on lung autopsy tissue using probe sets for *AGER* (to mark AT1 cells), *SFTPC* (to mark AT2 cells), and SARS-CoV-2 ORF1a RNA (Fig. 3a). We observed *AGER-* and *SFTPC*-high cells throughout the tissue (Fig. 3c). Reassuringly, we did not find many cells showing high levels of both genes (only 1 observed), allowing us to identify *AGER*- and *SFTPC*-high cells as AT1 and AT2 cells, respectively. Furthermore, the cells labeled by the *AGER* and *SFTPC* probe sets had morphologies consistent with AT1 and AT2 cells (see Fig. S7), respectively. For each marker gene, we selected a threshold number of mRNA molecules per cell to identify cells as either AT1 or AT2 cells (described in Materials and Methods). We then analyzed the fluorescence intensity of the ORF1a SARS-CoV-2 probe set signal across all 252,820 cells in the data set from patient 2 with high levels of infection in the lung. We found that a large fraction of the *SFTPC*-positive AT2 cells also stained as positive for ORF1a RNA (27.4%) but that none (0%) of the *AGER*-positive AT1 cells were positive for SARS-CoV-2 (Fig. 3b and d). There were also a substantial number of cells (647) that were ORF1a RNA positive but did not express either *SFTPC* or *AGER*. In addition, there were a number of cells with viral RNA staining that lacked nuclear DAPI staining suggesting that these represent dead cells that were previously infected (see Fig. S2b). In sum, we demonstrated highly specific discrimination of AT1 and AT2 cells and found that only the AT2 cell population had robust SARS-CoV-2 RNA consistent with these cells being the major target of infection in the lung.

10.1128/mbio.03751-21.7 FIG S7 Example images of AT2 and AT1 cell morphologies. We performed RNA FISH HCR with probe sets for AT2 marker *SFTPC* (a) and AT1 marker *AGER* (b) in lung samples from patients with SARS-CoV-2. AT2 cells are circled in yellow, and AT1 cells are circled in red. (c) AT2 cells are large and circular, while AT1 cells are thin and elongated. Scale bars of the large images indicate 25 μm. Inset images show examples of AT2 and AT1 cells in close proximity and have scale bars of 10 μm. Images were taken from large area scans of image tiles acquired at 20× magnification. Download FIG S7, PDF file, 0.2 MB. Copyright © 2022 Acheampong et al. 2022 Acheampong et al. https://creativecommons.org/licenses/by/4.0/ This content is distributed under the terms of the Creative Commons Attribution 4.0 International license.

**FIG 4:**
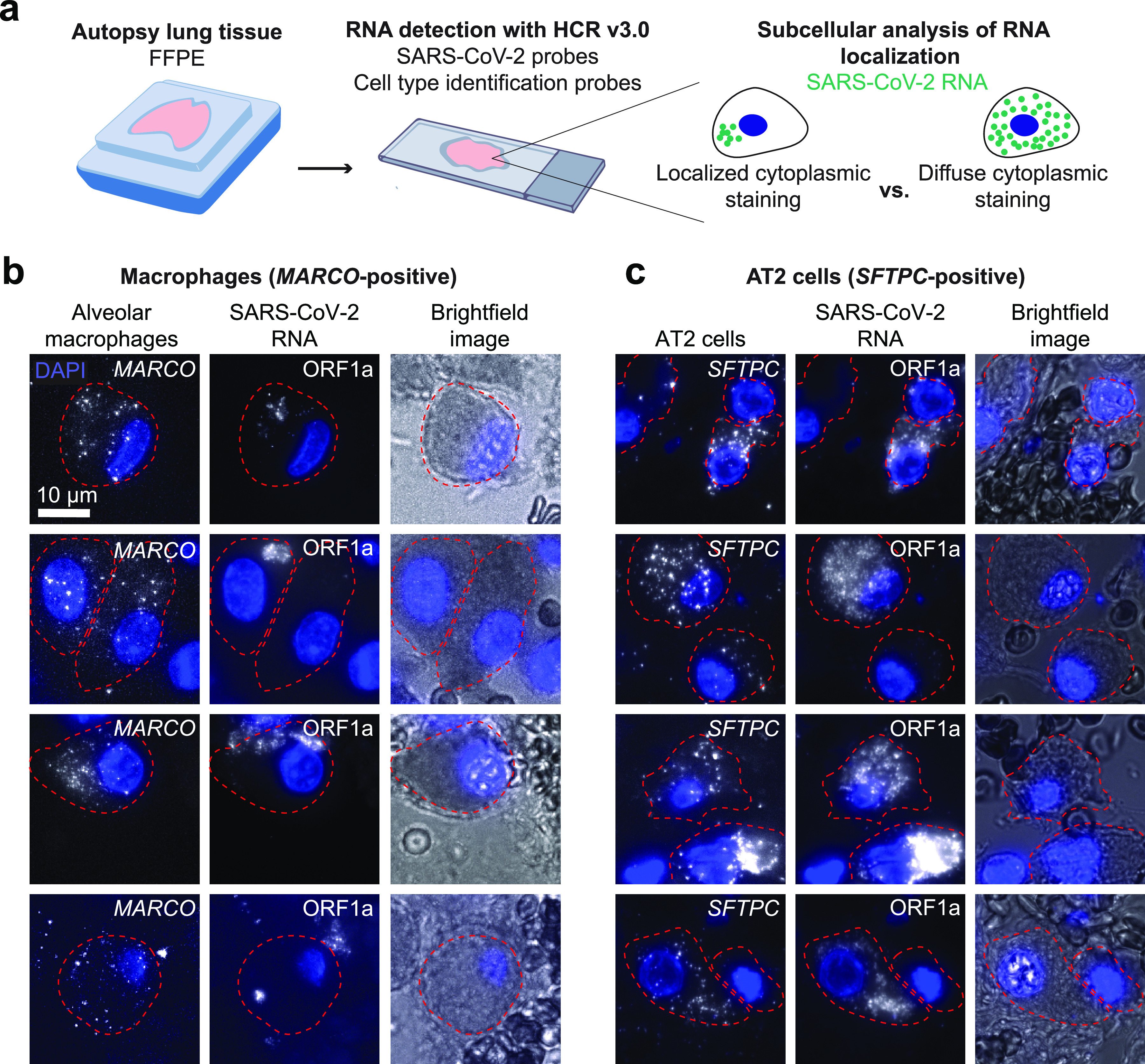
Alveolar macrophages and AT2 cells show distinct viral RNA staining patterns in autopsy tissue. (a) Schematic of experimental design in which we multiplexed cell-type-specific marker genes with SARS-CoV-2 ORF1a probes. We examined the subcellular distribution of RNA staining in infected alveolar macrophages and AT2 cells. (b) Examples of alveolar macrophages showing *MARCO*, ORF1a, and bright-field images for each cell. The border of each cell’s cytoplasm is shown by the red dotted line in each image. DAPI stain for cell nuclei is shown in blue. Scale bars show 10 μm. The images are z-projections of image stacks acquired at ×100 magnification. (c) Examples of AT2 cells showing *SFTPC*, ORF1a, and bright-field images for each cell. The borders, nuclei, and scale bars are labeled the same as in panel b. The images are z-projections of image stacks acquired at ×100 magnification.

We next sought to determine what other cell types contain SARS-CoV-2 RNA in the lung. We multiplexed a probe set for a macrophage-specific gene (*MARCO*) with the SARS-CoV-2 ORF1a probe set (Fig. 4a). We found a large number of *MARCO*-positive cells that contained staining with the SARS-CoV-2 ORF1a probe set (Fig. 4b). However, in many of these cells, the subcellular localization of the signal was distinct from the staining that we previously observed in *SFTPC*-positive AT2 cells (Fig. 4c). In many examples, the staining within alveolar macrophages appeared to be compartmentalized within a smaller region of the cytoplasm (compared to the AT2 cells). In AT2 cells, the ORF1a virus probe set typically stained the entire cell cytoplasm and around the entire periphery of the nucleus. It is possible that in the alveolar macrophages these smaller regions of staining represent restriction of the viral RNA to a subcellular compartment.

## DISCUSSION

In this paper, we outline methods and probe designs for visualizing SARS-CoV-2 RNA in cell lines and human autopsy specimens. First, to enable compatibility with autopsy tissue from COVID-19 patients, we developed a protocol for tissue processing (described in Materials and Methods). Next, to tailor the probes to the SARS-CoV-2 virus, we designed unique probe sets for the ORF1a and N region RNA. We further developed the assay to be able to uniquely label subgenomic mRNAs, which is not possible with conventional probe designs. We validated each of these probe sets in cell culture models and then applied them to autopsy tissues. We identified infected cells as those labeled by ORF1a RNA in lung, lymph node, and placenta.

In human lung tissue, we performed multiplex RNA FISH HCR with probe sets for cell-type-specific marker genes to determine that AT2 cells and alveolar macrophages contain SARS-CoV-2 RNA in the lung. Through subcellular visualization of the RNA localization, we found that the subcellular localization of ORF1a-containing transcripts is different between AT2 cells and alveolar macrophages. This finding further supports previous studies suggesting that alveolar macrophages acquire SARS-CoV-2 RNA through phagocytosis rather than receptor mediated entry (37–39). Furthermore, the observation that the viral RNA staining is confined to distinct regions of the cells could support other studies showing that alveolar macrophages contain viral RNA but do not produce replicating virus (40). Of note, this observation is based upon a single time point in infection, but the viral RNA could have different localization patterns throughout the course of disease. Additional studies are needed to fully capture how viral RNA localization changes in tissue across the time course of disease and in patients with different degrees of disease severity.

RNA FISH techniques have specific advantages over other tissue-based techniques for visualizing viruses. In the setting of a new viral threat, custom probes can be quickly designed to target the virus, only requiring knowledge of the viral sequence. Our probe design approach here ensures that the probes are specific only to the desired virus by querying sequence databases of other viruses. After designing the probe sequences, the synthesis of oligonucleotide probes is both inexpensive and fast such that the entire assay can easily be set up for a new virus in a relatively short period of time (3 to 5 days). As we do here, RNA FISH-based probes can be designed to different RNA species generated by the virus and even used to discriminate between closely related virus strains through both bioinformatic probe design strategies and different probe conformations. Examples include this study, in which we target both genomic RNAs and subgenomic mRNAs, as well as other studies with probes to positive and negative RNA strands (41), different segments of the influenza genome (21, 22), and even probes that detect single-base pair variants within the virus (22).

In addition to probes that target different components of the virus, we can also multiplex viral probes with probes for cellular genes. Here, we target cellular genes to identify cell types within the infected tissue, but this approach could be applied to profile other genes involved in the host response to viral infection. Furthermore, with recent advances in RNA *in situ* technologies it is now possible to probe hundreds to thousands of genes with techniques such as seqFISH+ and MERFISH (42, 43). These platforms could be easily adapted to include virus probes as well. Such an approach could reveal the full picture of how a viral infection alters a tissue, including the direct effects on the cells that are infected by the virus, as well as the effects on the neighboring cells and immune response.

The primary alternative methods for staining viruses in tissues rely on antibodies, including immunohistochemistry and immunofluorescence. In the setting of a pandemic with a new virus, the speed of antibody development, which can take weeks to months, can present significant challenges. Furthermore, antibody development can be costly and, even after production, antibodies still require extensive validation to prove that they are correctly targeting the protein of interest. In contrast, with modern sequencing-based epidemiologic surveillance, a novel agent’s genome may be available in days, and simple rules govern the design of suitable hybridization probes. Thus, it is less expensive, easier, and faster to develop RNA probes using oligonucleotides. Since IHC/IF and RNA FISH target different molecules (protein versus RNA, respectively), they also provide different and complementary information about the virus. With SARS-CoV-2, a number of studies have found viral protein staining by immunofluorescence or immunohistochemistry in different tissues but have not provided sufficient evidence to confirm that there is replicating virus present (11–13).

In summary, we outline here protocols and probe sets for using RNA FISH HCR in FFPE tissues for visualizing SARS-CoV-2 RNA. We demonstrate the use of these methods for visualizing different viral RNA species, identifying infected cells in FFPE tissues, and determining the cell types that are infected. Our work establishes RNA FISH HCR as a powerful technique for virology and pathology to visualize SARS-CoV-2 RNA in tissues that can be easily extended for new infectious diseases in the future.

## MATERIALS AND METHODS

### Human tissues.

Material from autopsies of patients who died of COVID-19 were obtained from family consented research only autopsies performed by the Department of Pathology and Laboratory Medicine at the Hospital of the University of Pennsylvania. Deidentified placenta samples were obtained through the Division of Anatomic Pathology at The Children’s Hospital of Philadelphia. Tissues were collected and formalin fixed for 48 to 72 h prior to routine processing and paraffin embedding. Tissues were sectioned to 5-μm thickness from FFPE blocks to be used in the ISH assays.

### Cell lines and infection.

We cultured A549^ACE2^ cells at 37°C and 5% CO2 in RPMI 1640 supplemented with 10% fetal bovine serum (FBS) and 1% penicillin-streptomycin. We cultured Huh7.5 cells at 37°C and 5% CO_2_ in Dulbecco modified Eagle medium (DMEM) supplemented with 10% FBS, 1% penicillin-streptomycin, and 1% L-GlutaMAX. For RNA *in situ* experiments, we seeded cells into two-well chambers (LabTek) at a density of 3,000 cells per well and then infected with SARS-CoV-2 (USA WA1/2020 strain) at an MOI of 1. SARS-CoV-2 was diluted in serum-free RPMI (for A549^ACE^) or DMEM (for Huh7.5) and added to cells for absorption for 1 h at 37°C. The inoculum was removed and replaced with RPMI with 2% FBS, and the cells were incubated at 37°C. We fixed the cells 24 h after infection in 4% formaldehyde and PBS for 30 min at room temperature. We then washed the sample with PBS two times and permeabilized them in 70% ethanol for up to 2 weeks before RNA FISH and RNA FISH HCR. All work with SARS-CoV-2 was performed in a biosafety level 3 laboratory using appropriate personal protective equipment and protocols approved by the Institutional Biosafety Committee and Environmental Health and Safety at the University of Pennsylvania.

### Probe design.

We designed RNA FISH HCR probes using RajLab ProbeDesignHD software (code freely available for noncommercial use here: https://github.com/arjunrajlaboratory/ProbeDesign/). This pipeline is implemented in MATLAB and uses a FASTA containing the RNA of interest. The software selects probe sequences according to length and free energy constraints and then excludes probes with complementarity to repetitive elements, human genome, and pseudogenes.

To target the SARS-CoV-2 genome, we referenced the sequence of the first U.S. isolate of SARS-CoV-2 (USA-WA1/2020) from the NCBI (GenBank MN985325.1). We used the probe designer described above to design nonoverlapping 52-mer oligonucleotides with a target Gibbs free energy for binding of −60 (allowable Gibbs free energy [−70, −50]) to the N and ORF1a regions of the SARS-CoV-2 genome, targeting only the 3,000- to 8,000-nucleotide region of the latter because it was the most conserved region among the strains circulating at the time as determined using nextstrain (44). We divided each 52-mer oligonucleotide into two nonoverlapping 25-mer sequences (removing the middle two nucleotides) and appended split-initiator HCR sequences using a custom MATLAB script (see Table S2 for probe sequences). For each probe, we then performed a local blast search against the human transcriptome and Nucleic Acids of Coronavirus and other Human Oronasopharynx pathogens (NACHO), a database we created that includes 562,446 sequences from other viruses that infect the human respiratory tract. All probes in the top 5% of hits based on E value and bit score were excluded, and the final probe sequences were synthesized from Eurofins at a nanomolar scale. Finally, we resuspended HCR probes to 100 μM in nuclease-free water and then combined these probes into pools each at a final concentration of 2 μM per probe. In the final probe designs, the ORF1a region probe set consisted of 23 probe pairs, and the N region probe set consisted of 7 probe pairs.

10.1128/mbio.03751-21.9 TABLE S2 RNA FISH HCR and RNA FISH probe oligonucleotide sequences. The table summarizes all oligonucleotide sequences used for RNA FISH HCR and single-molecule RNA FISH. Download Table S2, XLSX file, 0.01 MB. Copyright © 2022 Acheampong et al. 2022 Acheampong et al. https://creativecommons.org/licenses/by/4.0/ This content is distributed under the terms of the Creative Commons Attribution 4.0 International license.

To target SARS-CoV-2 subgenomic RNAs, we referenced the UCSC Genome Browser for SARS-CoV-2 genome data sets (https://genome.ucsc.edu/covid19.html) and RNA sequencing data sets (45) to identify the most frequent junction locations and peri-junction sequences based on the most abundant subgenomic RNA junction spanning reads. We then manually designed 52-mer oligonucleotides each spanning a unique leader-body junction for each of the eight canonical subgenomic RNAs generated via discontinuous transcription. We split each 52-mer oligonucleotide into two 25-mer sequences and appended split-initiator HCR sequences, as outlined earlier (see Table S2 for probe sequences). The final probes sequences were synthesized and resuspended the as described for the SARS-CoV-2 genome-targeting probes.

### Selection of cell-type-specific genes.

To identify individual cell types from human lung tissue, we reconciled RNA expression level data from multiple single-cell RNA sequencing data sets and identified genes that were both highly expressed and specific to one cell type (36). Full computational analysis scripts are available (https://drive.google.com/drive/folders/10sJ9Rhr5Z9stCP_ELZUQJr60ZvpE3dUR?usp=sharing). We then designed probes for specific cell types (see Table S2) similarly to our SARS-CoV-2 genomic probes and appended split-initiator HCR sequences using our custom MATLAB script. Final probe sequences were synthesized, resuspended, and then combined into pools, as outlined earlier.

### HCR RNA FISH.

We adapted the previously published HCR v3.0 protocol (24) for HCR RNA FISH in cultured cells as follows. We used 1.2 pmol each of our pooled HCR RNA FISH probe sets per 0.3 mL of hybridization buffer at 37°C. Our primary hybridization buffer consisted of 30% formamide, 10% dextran sulfate, 9 mM citric acid (pH 6.0), 50 μg mL^−1^ of heparin, 1× Denhardt solution (Invitrogen), and 0.1% Tween 20. For primary hybridization, we used 300 μL of hybridization buffer containing the appropriate probes per well of a two-well plate (Thermo Fisher Scientific), covered the well with a glass coverslip, and incubated the samples in containers humidified with 2× SSC at 37°C overnight (12 to 16 h). After the primary probe hybridization, we washed samples 4 × 5 min (i.e., four times for 5 min each time) at 37°C with wash buffer containing 30% formamide, 9 mM citric acid (pH 6.0), 50 μg mL^−1^ of heparin, and 0.1% Tween 20. We then washed the samples 2 × 5 min with 5× SSCT (5× SSC + 0.1% Tween 20) at room temperature and then incubated the samples at room temperature for 30 min in amplification buffer containing 10% dextran sulfate and 0.1% Tween 20. During this incubation, we snap-cooled 0.6 μL per well of individual 3 μM HCR hairpins (Molecular Instruments) conjugated to Alexa Fluor 647 (Alexa 647), Alexa Fluor 594 (Alexa 594), Alexa Fluor 546 (Alexa 546), or Alexa Fluor 488 (Alexa 488) in separate PCR tubes by heating at 95°C for 90 s and then either ramp cooling the sample at a ramp rate of 0.08°C/s to room temperature in 30 min or immediately transferring it to room temperature for 30 min concealed from light. Next, we pooled the hairpins in 300 μL of amplification buffer to a final concentration of 6 nM each. We added the hairpin solution to samples, placed a glass coverslip on top and then incubated samples at room temperature overnight (12 to 16 h) concealed from light. After hairpin amplification, we washed samples 5 × 5 min with 5× SSCT, added 100 μL of SlowFade antifade mounting solution containing 50 ng mL^−1^ of DAPI (Invitrogen) with a coverslip, and proceeded to image the samples.

For HCR RNA FISH on formalin-fixed paraffin-embedded (FFPE) tissues, we obtained tissues fixed via 10% neutral buffered formalin. We deparaffinized tissue sections on slides by first immersing them 2 × 10 min in xylene (Sigma-Aldrich) and then immersing them 2 × 5 min in 100% ethanol. We transferred the tissue slides to a 3:1 methanol-acetic acid solution at room temperature for 5 min, washed the slides in nuclease-free water for 3 min, and then performed antigen retrieval by placing the slides in a solution of 10 mM sodium citrate (pH 6) plus 0.1% diethyl pyrocarbonate (Sigma-Aldrich) heated with a 150°C water bath for 15 min. After antigen retrieval, we quickly rinsed the slides with 5× SSCT and immediately proceeded to HCR RNA FISH, which we adapted from the previously published HCR v3.0 protocol (24) for HCR RNA FISH in FFPE tissues as follows. We first prehybridized our samples by adding 200 μL of hybridization buffer warmed to 37°C and incubating the sample at 37°C for 10 min. While prehybridizing, we made our primary hybridization solution containing 0.8 pmol of each of our pooled HCR RNA FISH probes per 0.2 mL of hybridization buffer. Our primary hybridization buffer consisted of 30% formamide, 10% dextran sulfate, 9 mM citric acid (pH 6.0), 50 μg mL^−1^ of heparin, 1× Denhardt solution (Invitrogen), and 0.1% Tween 20. For primary hybridization, we used 50 to 100 μL of hybridization buffer containing the appropriate probes per slide, covered the section with a glass coverslip, and incubated the samples in humidified containers at 37°C overnight (12 to 16 h). After the primary probe hybridization, we washed the samples sequentially in 75% wash buffer (containing 30% formamide, 9 mM citric acid [pH 6.0], 50 μg mL^−1^ of heparin, and 0.1% Tween 20) plus 25% 5× SSCT (5× SSC + 0.1% Tween 20) solution, 50% wash buffer plus 50% 5× SSCT solution, 25% wash buffer plus 75% 5× SSCT solution, and 100% 5× SSCT for 15 min each at 37°C. We then washed the samples in 5× SSCT at room temperature for 5 min and incubated the samples at room temperature for 30 min in an amplification buffer containing 10% dextran sulfate and 0.1% Tween 20. During this incubation, we snap-cooled, by heating at 95°C for 90 s in separate PCR tubes, 0.2 μL per slide of individual 3 μM HCR hairpins (Molecular Instruments) conjugated to Alexa 647, Alexa 594, Alexa 546, or Alexa 488 and then either ramp-cooled the sample at a ramp rate of 0.08°C/s to room temperature in 30 min or immediately transferred the samples to room temperature to cool for 30 min concealed from light. After these 30-min treatments, we pooled the hairpins in 100 μL of amplification buffer per slide to a final concentration of 6 nM each. We added the hairpin solution to samples, placed a glass coverslip on top, and then incubated samples at room temperature overnight (12 to 16 h) concealed from light. After hairpin amplification, we washed samples 1 × 5 min in 5× SSCT, 2 × 15 min in 5× SSCT, and then 1 × 5 min with 5× SSCT again. We then stained nuclei by adding 200 μL of 5× SSCT containing 50 ng mL^−1^ of DAPI to the samples for 5 min at room temperature, quenched autofluorescence using the Vector TrueVIEW Autofluorescence Quenching kit according to the manufacturer’s protocol, added a coverslip, and then proceeded to image the samples. We note that the final hairpin concentrations used in these experiments are 10-fold lower than in the manufacturer’s protocol, which we optimized to reduce nonspecific amplification while still enabling sensitive RNA detection.

### Single-molecule RNA FISH.

We performed single-molecule RNA FISH according to existing protocols (23). We used a total of 38 oligonucleotides for ORF1a segment probes in Atto488 and 35 oligonucleotides for N segment probes in Cy3. Probe sequences for single-molecule RNA FISH are in Table S2.

### Imaging.

We imaged HCR RNA FISH samples on an inverted Nikon Ti2-E microscope equipped with a SOLA SE U-nIR light engine (Lumencor), an ORCA-Flash 4.0 V3 sCMOS camera (Hamamatsu), ×20 Plan-Apo λ (Nikon MRD00205), ×60 Plan-Apo λ (MRD01605), and ×100 Plan-Apo λ (MRD01905) objectives and filter sets for DAPI, Alexa Fluor 488, Alexa Fluor 594, and Atto647N. Our exposure times ranged from 100 to 200 ms for most of the dyes except for DAPI, for which we used ∼50-ms exposures. For RNA FISH HCR cell culture experiments in Fig. 1, we acquired z-stack images using 50- to 100-ms exposure times. For the experiments depicted in Fig. 2 and 4, we first acquired tiled images in a single *z*-plane (scan) at ×20 magnification, from which we identified positions containing cells positive for SARS-CoV-2 and returned to those positions to acquire a *z*-stack at ×60 or ×100 magnification. For large area scans, we used Nikon Perfect Focus to maintain focus across the imaging area. For the single-molecule RNA FISH experiments in Fig. S1, we acquired z-stack images with 300- to 500-ms exposure times using green fluorescent protein (GFP) and Cy3.

### Image analysis.

For quantifying fluorescence intensity in cell culture samples in Fig. 1, we used custom MATLAB scripts available at https://github.com/arjunrajlaboratory/rajlabimagetools. Briefly, our image analysis consisted of manual segmentation of the boundaries for each cell and then quantification of the total fluorescence intensity within that boundary. For the plotting in Fig. 1, we normalized the total fluorescence intensity across all pixels in the cell to the total cell area.

For tissue image analysis, we first developed a custom MATLAB pipeline for cropping tiled, single *z*-plane 20 × 20 scan images taken at ×20 magnification into smaller images. We then used CellProfiler to segment cells using 4′,6′-diamidino-2-phenylindole (DAPI) to identify nuclei. We dilated the nuclear objects by a radius of 6 pixels, 7 pixels, and 6 pixels for lung tissue, hilar lymph node tissue, and placenta tissue, respectively, to capture approximately the diameter of one whole cell in the tissue. We measured the position and intensities of the fluorescence signal for each of the SARS-CoV-2 probe sets in each cell. We excluded cells touching image borders. For each cell, we determined the intensity of the SARS-CoV-2 ORF1a probe set by using the cutoff for the upper quartile of pixel intensities across the area of the cell. This processing was necessary because many cells did not have staining throughout the cell area. The code to run this analysis in CellProfiler is available at https://drive.google.com/drive/folders/10sJ9Rhr5Z9stCP_ELZUQJr60ZvpE3dUR?usp=sharing.

To determine the fluorescence intensity threshold to label a cell as SARS-CoV-2 positive (in Fig. 2 and 3), we adapted methods from Pereira et al. (46). Briefly, we normalized the upper quartile intensity measurements of SARS-CoV-2 ORF1a staining (Alexa 647 channel) in each cell to the median intensity across all cells. We then log-transformed the median-normalized data and used the MClust function in R to fit a two-state lognormal mixture model with unequal variance. We evaluated the model fit by an *F* test and selected an appropriate intensity gate that captured only the positive cells in the leftmost distribution. In exposure-matched negative-control samples, we found that our *F* test returned insignificant *P* values (*P* > 0.05), indicating the presence of only one population of cells with the baseline of background staining. Our intensity gates did not capture any cells in our negative-control samples.

For analyses in which we used our SARS-CoV-2 ORF1a probe set with Alexa 488 fluorescent hairpins (Fig. 3), we needed a way to exclude the autofluorescent background in that channel from our analysis. To do this, we acquired images with a different filter set for which we did not have any dye in the experiment (Alexa 594). We then analyzed these images to identify cells with high levels of nonspecific signal in this wavelength using the same approach as described for the SARS-CoV-2 ORF1a analysis above. We set a threshold intensity for which cells above had high nonspecific signals and removed these cells from our analysis. After removing these cells from the data set, we proceeded to analyze the ORF1a GFP signal using the analysis pipeline as for Alexa647 described above.

For infected cell type identification analyses shown in Fig. 3, we cropped the large image scans down to individual tiles consisting of roughly one field of view and then segmented the cells as described above using CellProfiler. We dilated the nuclear segments by a radius of 6 pixels to capture the entire area of each cell. Within each cell, we used the “enhance features” module in CellProfiler to enhance the signal (Alexa 647 and Alexa 546 channels) from the single-molecule HCR probes for cell-type-specific genes. We then set a threshold for calling individual HCR spots and assigned the number of spots to each cell. We determined the cell type by plotting the distribution of spot counts for each cell type marker and selecting a threshold that captured the tails of the distributions and adjusted these thresholds manually by referencing HCR RNA FISH images to ensure that our thresholds were reasonably accurate. The threshold for *AGER* was 7 spots (to identify a cell as AT1) and the threshold for *SFTPC* was 6 (to identify a cell as an AT2 cell). Cells that did not meet thresholds or could not be classified based on our parameters were assigned as undetermined.

### Data availability.

All data and remaining code for these analyses can be found at https://drive.google.com/drive/folders/10sJ9Rhr5Z9stCP_ELZUQJr60ZvpE3dUR?usp=sharing or, upon reasonable request, obtained from the corresponding author. All analyses were done in R, MATLAB, or CellProfiler.
