# Supplementary material for: Subcellular Detection of SARS-CoV-2 RNA in Human Tissue Reveals Distinct Localization in Alveolar Type 2 Pneumocytes and Alveolar Macrophages
Source: mBio. 2022 Feb 8;13(1):e03751-21. doi: 10.1128/mbio.03751-21 (PMC8822351; doi:10.1128/mbio.03751-21)
Supplement: FIG S1 [file mbio.03751-21-sf001.pdf]

Supplementary Figure 1

Single-molecule RNA FISH for SARS-CoV-2 (ORF1a and N)

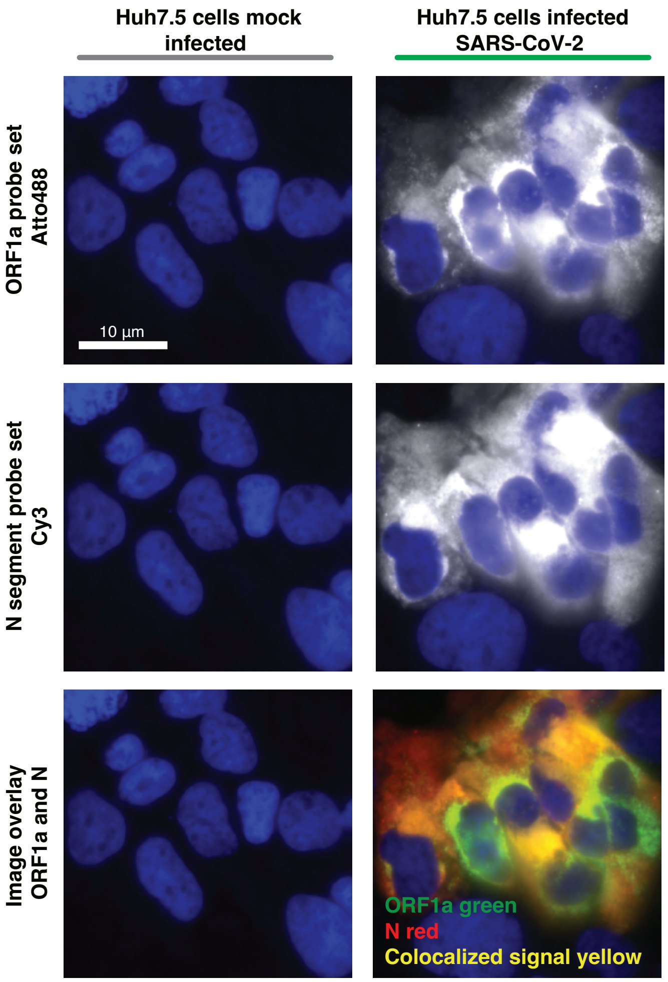

Supplementary Figure 1: Single-molecule RNA FISH with probes targeting ORF1a and N regions in Huh7.5 cells infected with SARS-CoV-2. Representative images of Huh7.5 cells infected with SARS-CoV-2 and hybridized with RNA FISH probes targeting ORF1a and N. Similar to the RNA FISH HCR, we observed higher fluorescence signal intensity in the perinuclear region of the ORF1a probe compared to the N probe. The bottom row of images is a composite of the ORF1a probe (green), N probe (red), and DAPI signal. In all images, the DAPI stain for cell nuclei is shown in blue. Scale bars are 10 µm. The images are z-projections from image stacks acquired at 60X.
