# Supplementary material for: Subcellular Detection of SARS-CoV-2 RNA in Human Tissue Reveals Distinct Localization in Alveolar Type 2 Pneumocytes and Alveolar Macrophages
Source: mBio. 2022 Feb 8;13(1):e03751-21. doi: 10.1128/mbio.03751-21 (PMC8822351; doi:10.1128/mbio.03751-21)
Supplement: FIG S2 [file mbio.03751-21-sf002.pdf]

Supplementary Figure 2

a **Regions of viral staining without nuclei**

SARS-CoV-2 genomic RNA

Brightfield image

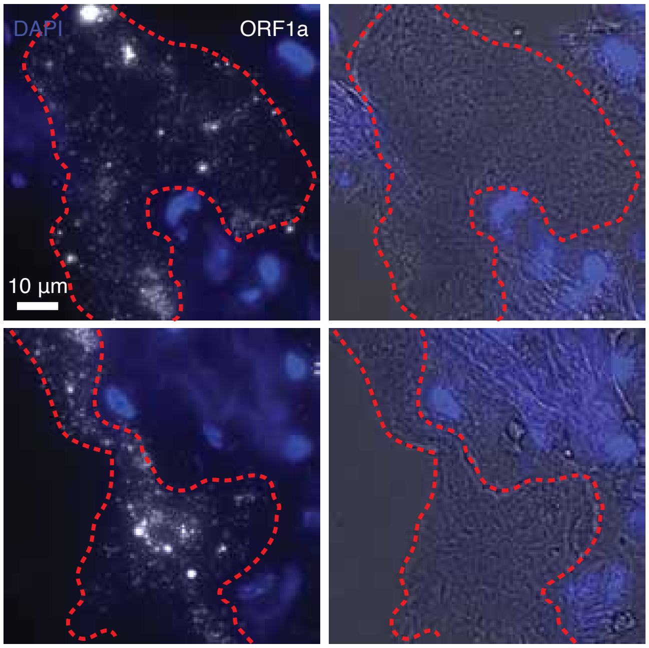

b **Examples of smaller discrete regions of viral RNA without nuclei**

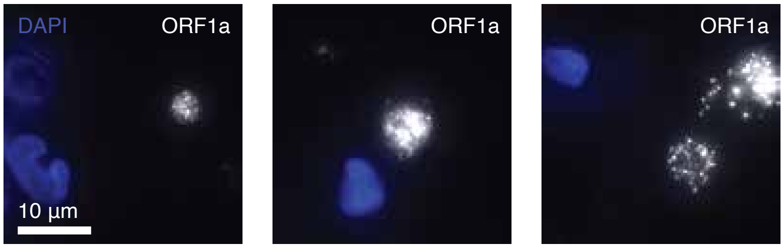

Supplementary Figure 2: Examples of regions in the lung tissue that show viral staining with ORF1a probes, but do not have nuclei. A. Two example regions in which we observed extensive viral staining with the ORF1a probe set, but did not observe DAPI signal. Right image is RNA FISH HCR for ORF1a and the left is the brightfield image. Red dotted lines show areas of interest with ORF1a staining. B. Examples of small discrete regions of ORF1a staining without DAPI staining. In all images, the DAPI stain for cell nuclei is shown in blue. Scale bars are 10 µm. The images are z-projections of image stacks acquired at 100X magnification.
