# Supplementary material for: Subcellular Detection of SARS-CoV-2 RNA in Human Tissue Reveals Distinct Localization in Alveolar Type 2 Pneumocytes and Alveolar Macrophages
Source: mBio. 2022 Feb 8;13(1):e03751-21. doi: 10.1128/mbio.03751-21 (PMC8822351; doi:10.1128/mbio.03751-21)
Supplement: FIG S3 [file mbio.03751-21-sf003.pdf]

Supplementary Figure 3

Computational identification of infected cells in each tissue

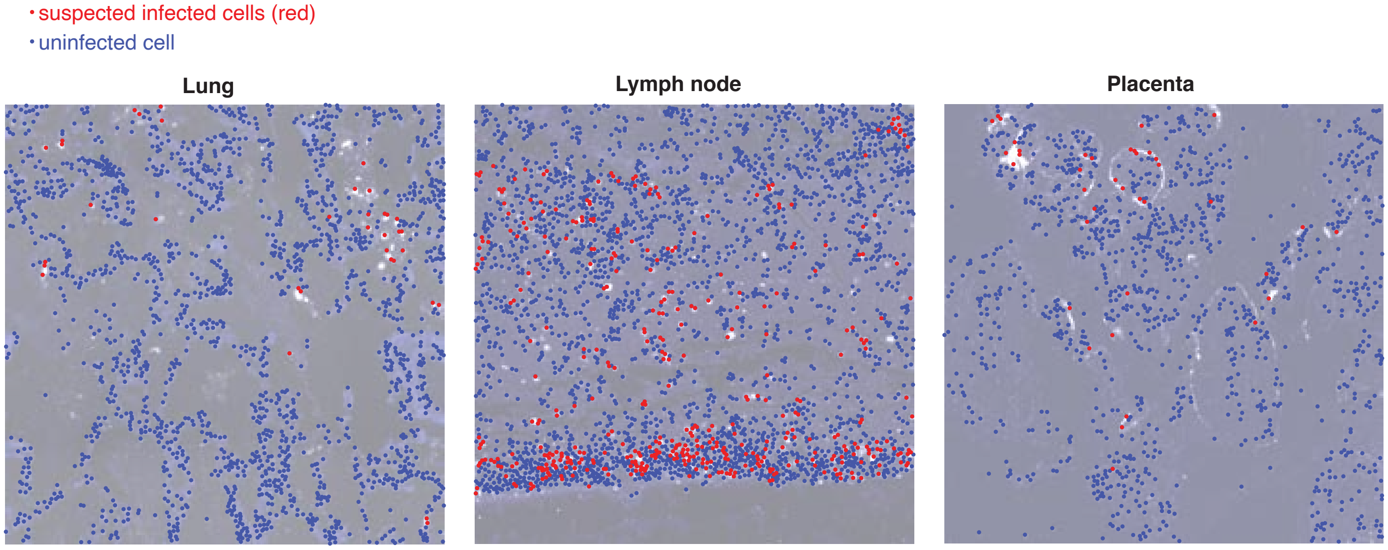

n=1,766 cells displayed
69,274 cells total in dataset

n=2,871 cells displayed
311,385 cells total in dataset

n=1,119 cells displayed
33,420 cells total in dataset

Supplementary Figure 3: Computational analysis identifying suspected infected cells in each tissue. Images overlayed with the results from our image processing pipeline. Blue dots label cells with fluorescence signal below the cutoff for infected and red dots label cells above the cutoff for infected. Images are from a subset of the data. Total number of cells displayed in each region and the total number of cells in each data set is below each image. Images are large area scans of individual image tiles acquired at 20X magnification.
