# Supplementary material for: Subcellular Detection of SARS-CoV-2 RNA in Human Tissue Reveals Distinct Localization in Alveolar Type 2 Pneumocytes and Alveolar Macrophages
Source: mBio. 2022 Feb 8;13(1):e03751-21. doi: 10.1128/mbio.03751-21 (PMC8822351; doi:10.1128/mbio.03751-21)
Supplement: FIG S4 [file mbio.03751-21-sf004.pdf]

Supplementary Figure 4

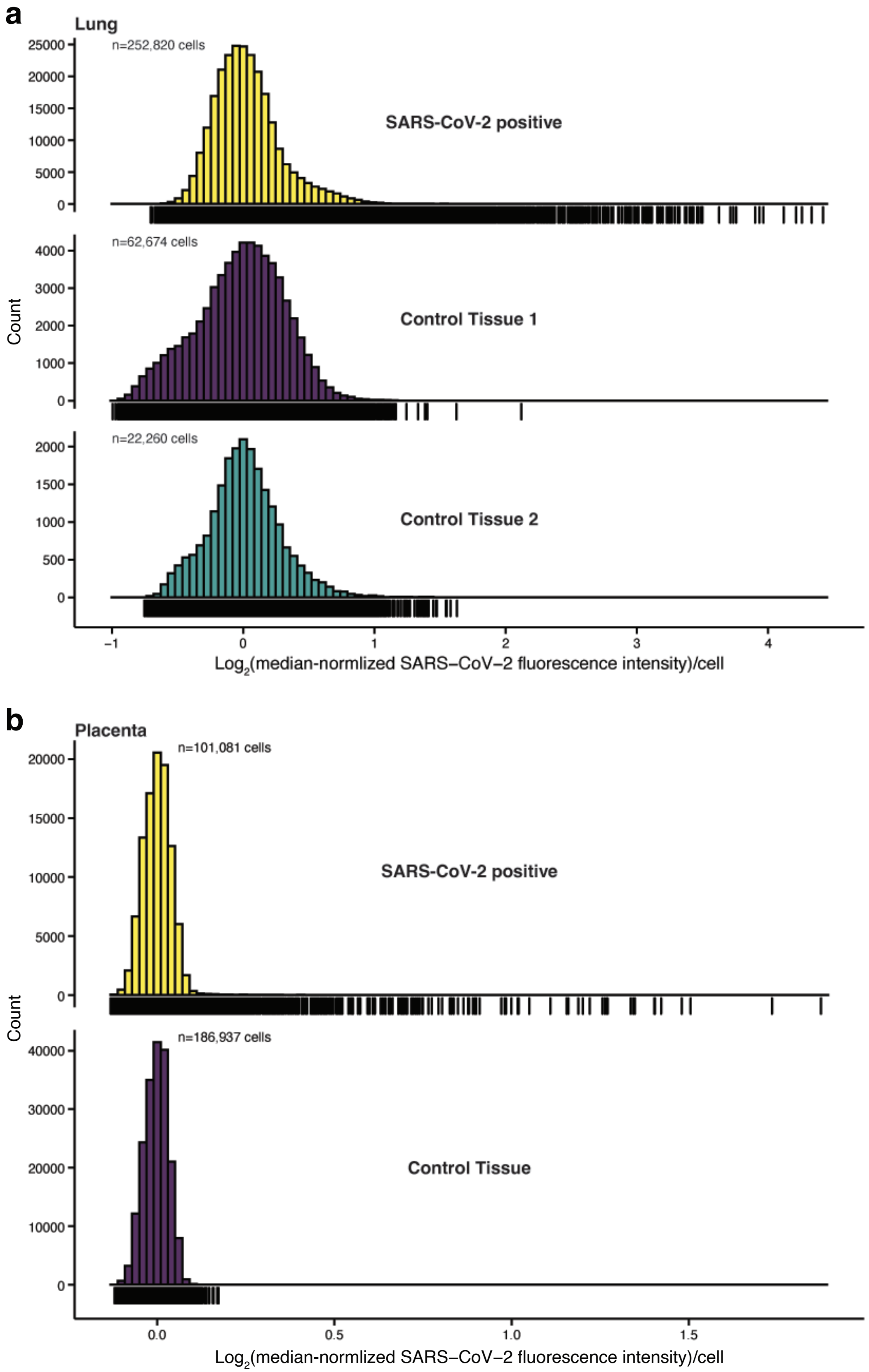

Supplementary Figure 4: Comparison of infected tissue to control tissues samples in which the patients did not have SARS-CoV-2 infection. We performed RNA FISH HCR in a. lung and b. placenta samples from patients with SARS-CoV-2 infection (yellow) and control samples from patients that did not have SARS-CoV-2 infection (purple and teal). Histograms display the Log2 transformation of median normalized ORF1a fluorescence signal in each cell. Infected samples have much higher median-normalized ORF1a fluorescence signal than control tissue. The distribution of median-normalized ORF1a fluorescence signal is significantly different in infected samples compared to control samples where the infected samples have a tail made up of infected cells with much higher signal than control tissue in both the lung (single-tailed KS test, p-value < 2.2e-16 when compared to control sample 1 and p-value < 2.2e-16 when compared to control sample 2) and the placenta (single-tailed KS test, p-value < 2.2e-16).
