# Supplementary material for: Subcellular Detection of SARS-CoV-2 RNA in Human Tissue Reveals Distinct Localization in Alveolar Type 2 Pneumocytes and Alveolar Macrophages
Source: mBio. 2022 Feb 8;13(1):e03751-21. doi: 10.1128/mbio.03751-21 (PMC8822351; doi:10.1128/mbio.03751-21)
Supplement: FIG S5 [file mbio.03751-21-sf005.pdf]

Supplementary Figure 5

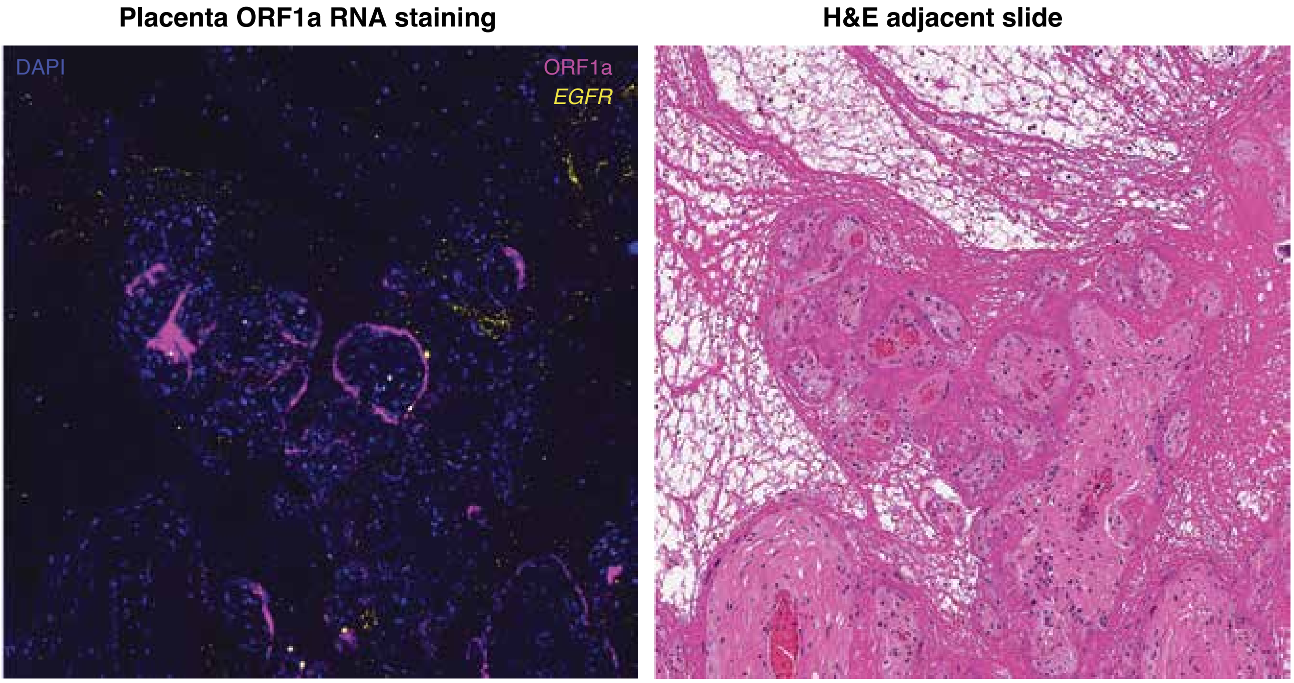

Supplementary Figure 5: Example region of placenta with RNA FISH HCR for ORF1a with an adjacent tissue section stained with H&E. We performed RNA FISH HCR with probe sets for ORF1a and EGFR. On the adjacent section, we stained the tissue with hematoxylin and eosin. We took tiled image scans of the fluorescence slide and used a slide scanner for the H&E. We aligned the two images to identify the corresponding H&E region for which we found cells staining with the ORF1a probe set. ORF1a fluorescence signal is in pink, EGFR is in yellow, and DAPI is in blue. Images are large area scans of image tiles acquired at 20X magnification.
