# Supplementary material for: Subcellular Detection of SARS-CoV-2 RNA in Human Tissue Reveals Distinct Localization in Alveolar Type 2 Pneumocytes and Alveolar Macrophages
Source: mBio. 2022 Feb 8;13(1):e03751-21. doi: 10.1128/mbio.03751-21 (PMC8822351; doi:10.1128/mbio.03751-21)
Supplement: FIG S6 [file mbio.03751-21-sf006.pdf]

Supplementary Figure 6

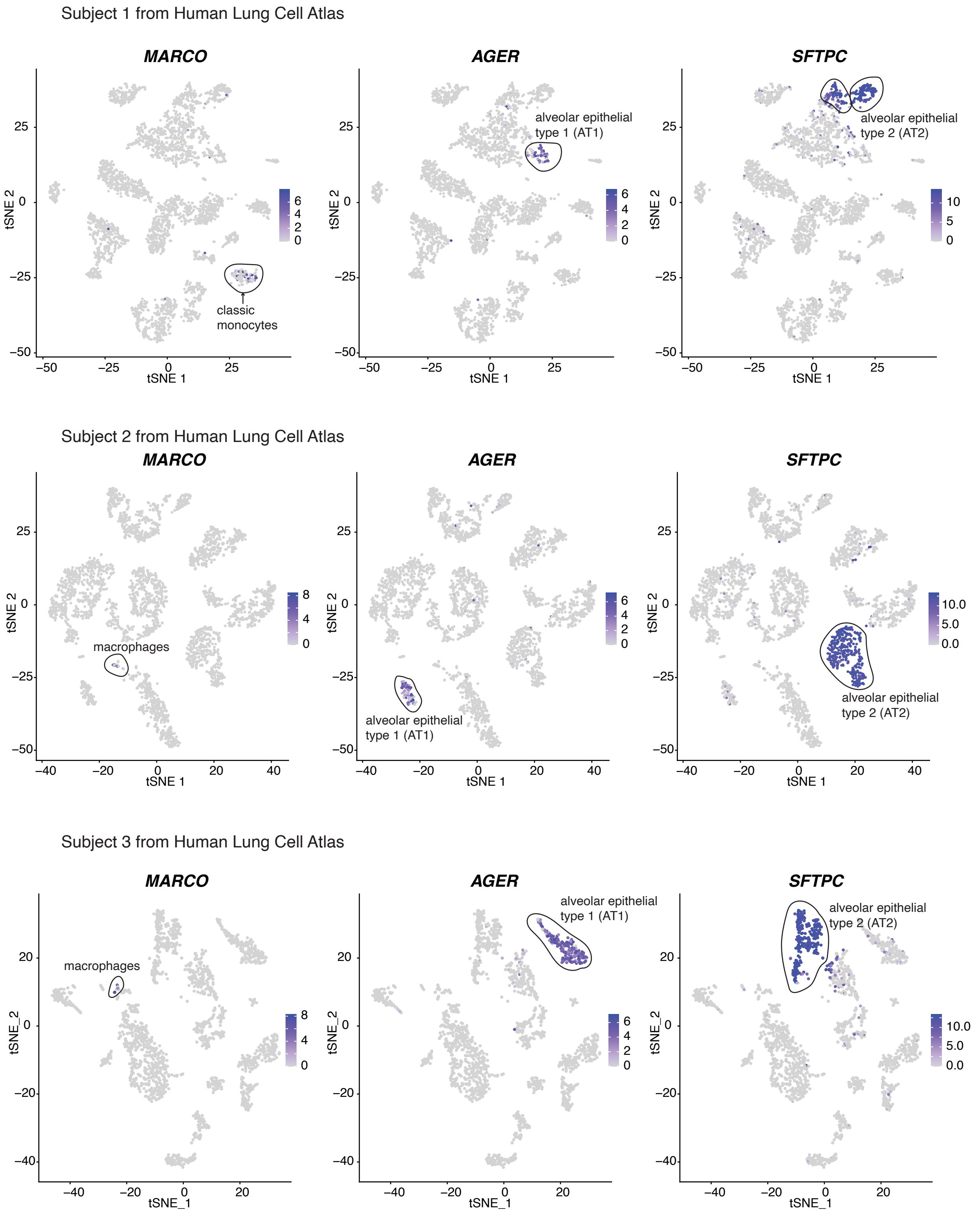

Supplementary Figure 6: tSNE plots of the human lung cell atlas single-cell RNA-sequencing data across 3 subjects. Each plot is a tSNE projection of all cells in the data set with the color of the points depicting the expression of the gene. Each row of plots is from a different subject. The target cell-type with each marker is labeled on the plots with a circle around the cluster (monocytes/macrophages, AT1 cells, AT2 cells). The three genes identified as cell-type specific markers are *MARCO*, *AGER*, and *SFTPC*.
