# Supplementary material for: Subcellular Detection of SARS-CoV-2 RNA in Human Tissue Reveals Distinct Localization in Alveolar Type 2 Pneumocytes and Alveolar Macrophages
Source: mBio. 2022 Feb 8;13(1):e03751-21. doi: 10.1128/mbio.03751-21 (PMC8822351; doi:10.1128/mbio.03751-21)
Supplement: FIG S7 [file mbio.03751-21-sf007.pdf]

Supplementary Figure 7

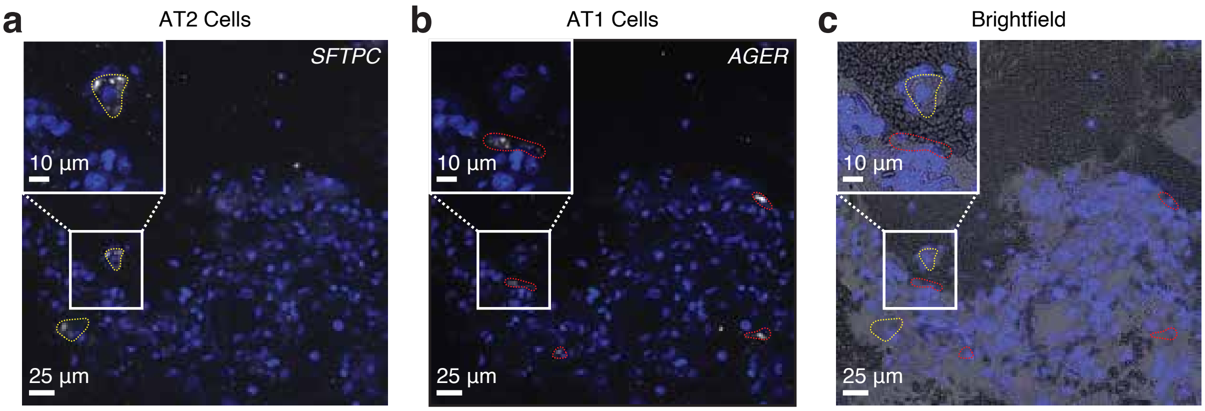

Supplementary Figure 7: Example images of AT2 and AT1 cell morphologies. We performed RNA FISH HCR with probe sets for **a.** AT2 marker SFTPC and **b.** AT1 marker AGER in lung samples from patients with SARS-CoV-2. AT2 cells are circled in yellow and AT1 cells are circled in red. **c.** AT2 cells are large and circular while AT1 cells are thin and elongated. Scale bars of the large images are 25 µm. Inset images show examples of AT2 and AT1 cells in close proximity and have scale bars of 10 µm. Images were taken from large area scans of image tiles acquired at 20X magnification.
